# Relationship Between Mitral Annular Calcification and Inflammatory Indices in Patients with Cardiometabolic Risk Factors

**DOI:** 10.3390/biomedicines14020398

**Published:** 2026-02-09

**Authors:** Paula Cristina Morariu, Alexandru Florinel Oancea, Maria Mihaela Godun, Diana Elena Floria, Oana Sîrbu, Anca Ouatu, Daniela Maria Tanase, Ionela Daniela Morariu, Cristina Gena Dascălu, Mariana Floria

**Affiliations:** 1 Department of Internal Medicine, Faculty of Medicine, Grigore T. Popa University of Medicine and Pharmacy, 700115 Iasi, Romania; morariu.paula-cristina@email.umfiasi.ro (P.C.M.); alexandru.oancea@umfiasi.ro (A.F.O.); godun.maria-mihaela@email.umfiasi.ro (M.M.G.); diana-elena.iov@d.umfiasi.ro (D.E.F.); anca.ouatu@umfiasi.ro (A.O.); daniela.tanase@umfiasi.ro (D.M.T.); floria.mariana@umfiasi.ro (M.F.); 2 Faculty of Pharmacy, Grigore T. Popa University of Medicine and Pharmacy, 700115 Iasi, Romania; 3 Department of Medical Informatics and Biostatistics, Faculty of Dental Medicine, Grigore T. Popa University of Medicine and Pharmacy, 700115 Iasi, Romania; cristina.dascalu@umfiasi.ro

**Keywords:** mitral annular calcification, inflammation, cardiovascular disease, cardiometabolic syndrome, diabetes, prediabetes, neutrophil-to-lymphocyte ratio

## Abstract

**Background:** Mitral annular calcification (MAC) is associated with systemic atherosclerosis and cardiometabolic risk factors. Although hematologic inflammatory indices have been reported to be correlated with MAC, whether these associations persist after accounting for the cardiometabolic context in which MAC occurs remains unclear. **Methods:** In a prospective, cross-sectional study of consecutive adults, patients with mild MAC were compared to those without MAC. Individuals with major inflammatory conditions, advanced chronic kidney disease, cirrhosis, malignancy, autoimmune/acute inflammatory disorders, significant valvular disease, prosthetic valves/pacing devices, psychiatric disorders, or moderate-severe MAC were excluded. C-reactive protein (CRP) and hematological inflammatory indices, including neutrophil-to-lymphocyte ratio (NLR), Systemic Inflammatory Response Index (SIRI), and lymphocyte-to-leukocyte ratio (LLR), were analyzed in relation to MAC status. **Results:** Among 205 patients, 134 had mild MAC and 71 had no MAC. Patients with MAC were older and displayed higher cardiometabolic burden, including more frequent dysglycemia, higher blood pressure, and greater adiposity. In unadjusted comparisons, inflammatory markers differed according to MAC status: CRP (0.31 mg/dL vs. 0.18 mg/dL, *p* = 0.002), NLR (2.52 vs. 1.99, *p* = 0.032), SIRI (1.27 vs. 1.04, *p* = 0.039), and LLR (0.26 vs. 0.29, *p* = 0.032). In multivariable logistic regression models, none of the inflammatory markers remained independently associated with MAC. In contrast, age (ORs 1.056–1.063 per year increase, *p* ≤ 0.001), prediabetes (ORs 2.43–3.63, *p* ≤ 0.001), and type 2 diabetes (OR 5.91 and 6.19, *p* ≤ 0.001) demonstrated consistent independent associations with MAC across all models. **Conclusions:** In this cardiometabolic population with mild MAC, inflammatory indices showed unadjusted differences but no independent associations with MAC after comprehensive cardiometabolic adjustment. These findings are most compatible with inflammatory markers primarily reflecting the cardiometabolic milieu in which MAC occurs rather than representing MAC-specific processes. Age and glucose metabolism abnormalities emerged as the dominant independent factors associated with mild MAC, reinforcing the central role of metabolic dysfunction in MAC pathogenesis.

## 1. Introduction

Mitral annular calcification (MAC) is now regarded as a systemic pathological entity rather than a purely localized degenerative alteration [1,2,3]. It shows a strong association with generalized atherosclerotic disease, sharing key cardiovascular risk factors age, obesity, type 2 diabetes (T2D), dyslipidemia, and arterial hypertension. Evidence from pathological and clinical investigations indicates that MAC frequently coexists with carotid, coronary, and aortic atherosclerosis, suggesting the concept that MAC reflects a cardiac manifestation of systemic vascular disease [4,5,6,7,8,9].

Systemic inflammation (SI) represents a central mechanism linking traditional cardiovascular risk factors to atherosclerosis and may also contribute to the development of MAC by promoting leukocyte recruitment, cytokine release, and vascular smooth muscle cell apoptosis, which together facilitate calcium deposition within the fibrous cardiac skeleton [10,11]. Nonetheless, available data remains inconclusive regarding the causal role of inflammation in the development of MAC, the possibility that MAC itself induces a systemic inflammatory response, or whether both phenomena primarily reflect the influence of common cardiometabolic risk factors [12].

In this framework, readily available hematological indices of inflammation, including neutrophil-to-lymphocyte ratio (NLR) and the platelet-to-lymphocyte ratio (PLR), have attracted growing interest because of their low cost, ease of assessment, and recognized ability to reflect chronic low-grade systemic inflammation. NLR has been constantly associated with increased cardiovascular morbidity, the presence of arterial calcification, and unfavorable clinical outcomes across diverse patient populations [10,13,14]. Other composite scores like Systemic Inflammation Response Index (SIRI) and the Systemic Immune-Inflammation Index (SII) have been linked to atherosclerosis and cardiometabolic risk. These markers capture low-grade, subclinical SI using routinely available parameters and are increasingly used in cardiovascular research [15,16,17,18].

Although prior studies have reported associations between MAC and inflammatory markers such as NLR and PLR, these findings were largely derived from populations characterized by advanced age and a high burden of cardiometabolic comorbidities, conditions linked to chronic low-grade inflammation [10,19]. Consequently, it remains uncertain whether the reported associations are attributable to inflammatory mechanisms directly related to MAC or merely reflect the inflammatory milieu characteristic of cardiometabolic disease. In this setting, the present study was designed to examine the relationship between MAC and CRP and selected hematological inflammatory indices (including NLR, SIRI, and LLR) among patients with cardiometabolic factors, within the cardiometabolic context in which MAC develops.

## 2. Materials and Methods

### 2.1. Study Population and Design

This was a prospective, cross-sectional observational study conducted in the Internal Medicine Clinic of Saint Spiridon County Clinical Emergency Hospital, Iasi, Romania. During the study period (March 2023–August 2024), all consecutive adult patients (≥18 years) attending the clinic for cardiology consultation routinely underwent transthoracic echocardiography as part of standard care, regardless of the underlying diagnosis. From this consecutive series, patients were initially screened for the presence or absence of MAC on two-dimensional echocardiography.

Patients were excluded if they were unable to provide informed consent or if they had hemodynamically significant valvular heart disease (>moderate mitral or aortic regurgitation or stenosis), prosthetic heart valves, temporary or permanent cardiac pacing devices, or a documented psychiatric disorder. To reduce the confounding impact of major SI conditions on hematologic indices and to better characterize low-grade inflammation, individuals with advanced chronic kidney disease (<30 mL/min/1.73 m^2^), liver cirrhosis, active malignancy, chronic hematologic disorders, acute inflammatory conditions, or autoimmune diseases were also excluded. Patients with moderate to severe MAC were not included to ensure a homogeneous study population and avoid the potential hemodynamic consequences of advanced annular calcification on inflammatory markers. Accordingly, the analysis was restricted to patients with mild MAC and those without MAC, allowing a focused evaluation of the relation between early MAC and hematological inflammatory indices in a cardiometabolic risk context.

### 2.2. Clinical and Laboratory Assessment

Participants underwent a standardized clinical evaluation, including demographic information (age, sex, residential area), cardiovascular history, comorbidities (hypertension, T2D, prediabetes, dyslipidemia, hepatic steatosis, heart failure, atrial fibrillation, prior myocardial infarction, prior stroke), and lifestyle factors (smoking status).

Fasting venous blood samples were obtained from the antecubital vein after a 12 h fast using standard sterile technique. Hematological parameters were measured from EDTA-anticoagulated samples, while biochemical analyses were performed using routine laboratory methods. Renal function was assessed by estimating glomerular filtration rate (eGFR) using the CKD-EPI equation.

Inflammation-related indices were derived from complete blood count parameters, including NLR, PLR, neutrophil-to-monocyte ratio (NMR), and lymphocyte-to-leukocyte ratio (LLR). Two composite indices were also calculated: SIRI, calculated as (neutrophils × monocytes)/lymphocytes, and SII, defined as (neutrophils × platelets)/lymphocytes. These indices were selected due to their availability from routine testing and their established use as markers of low-grade SI in cardiovascular and cardiometabolic research.

Heart failure was defined according to ESC criteria (typical symptoms/signs, elevated natriuretic peptides, and structural or functional cardiac abnormalities) [20]. Both preserved ejection fraction and reduced ejection fraction phenotypes were included. Glucose metabolism status was defined according to contemporary guidelines, with T2D diagnosed by a documented history, fasting plasma glucose ≥ 126 mg/dL, HbA1c ≥ 6.5%, or glucose-lowering treatment. Prediabetes was defined as fasting plasma glucose 100–125 mg/dL and/or HbA1c 5.7–6.4%, in the absence of antidiabetic treatment. Hepatic steatosis was diagnosed by abdominal ultrasonography, based on increased liver echogenicity compared with the renal cortex and vascular blurring.

### 2.3. Echocardiographic Evaluation and MAC Definition

All patients underwent comprehensive transthoracic echocardiographic evaluation using a Samsung ultrasound system (Samsung Medison, Seoul, Republic of Korea) in accordance with current recommendations. Standard M-mode, two-dimensional, and Doppler imaging techniques were applied. Left ventricular end-systolic and end-diastolic volumes, as well as ejection fraction (LVEF), were calculated using the biplane Simpson’s method from apical four-chamber and apical two-chamber views. Left atrial volumes were assessed according to current echocardiographic recommendations. Valvular regurgitation and stenosis were evaluated using color Doppler imaging, complemented by pulsed- and continuous-wave Doppler recordings.

All examinations were performed by a single experienced operator to minimize interobserver variability, and each measurement was obtained twice in the same view to reduce intraobserver variability.

MAC was defined as a localized, highly echogenic structure at the junction between the atrioventricular groove and the base of the mitral valve leaflets, visualized consistently in the parasternal long-axis, parasternal short-axis, and apical four-chamber views. Only mild MAC was considered for analysis, defined as a focal calcific deposit with maximal thickness < 5 mm, involving less than 1/3 of the annular circumference and confined to the annulus, without extension into the left ventricular inflow tract or onto the leaflets or adjacent myocardium [21]. Calcification thickness was measured from the leading to the trailing edge at the site of greatest echo-density.

### 2.4. Ethical Considerations

The study protocol was approved by the Ethics Committee of the Saint Spiridon Emergency Clinical Hospital (no. 28/9 March 2023) and by the Ethics Committee of the Grigore T. Popa University of Medicine and Pharmacy, Iasi (no. 303/16 May 2023). All participants provided informed consent prior to enrollment, in accordance with the principles of the Declaration of Helsinki.

### 2.5. Statistical Analysis

Statistical analyses were performed using IBM SPSS Statistics version 29.0 (IBM Corp., Armonk, NY, USA). Data distribution was assessed using the Shapiro-Wilk test. Continuous variables are presented as mean ± standard deviation for normally distributed variables and as median with interquartile range (IQR) for non-normally distributed variables. Categorical variables are expressed as frequencies and percentages. Group comparisons between patients with and without MAC were conducted using appropriate parametric and non-parametric tests: the independent samples *t*-test for normally distributed continuous variables and the Mann-Whitney U test for non-normal variables. Categorical variables were compared using the chi-square (χ^2^) test. Trends toward significance were noted for *p*-values between 0.05 and 0.10. Receiver operating characteristic (ROC) curve analysis was used to descriptively assess the ability of inflammatory markers to differentiate between patients with and without MAC, with calculation of the area under the curve (AUC) and corresponding 95% confidence intervals (CIs). Correlations between inflammatory markers and selected clinical and cardiometabolic variables were evaluated using Spearman’s rank correlation coefficients. Multivariable binary logistic regression analyses were performed to assess the association between inflammatory markers and MAC after accounting for relevant cardiometabolic and clinic covariates. To avoid multicollinearity between mathematically related inflammatory indices, separate regression models were constructed, each including only one inflammatory marker at a time. Results are reported as odds ratios (ORs) with 95% confidence intervals (CIs). Model calibration was assessed using the Hosmer-Lemeshow goodness-of-fit test. All tests were two-sided, and a *p*-value < 0.05 was considered statistically significant.

## 3. Results

**Figure 1:**
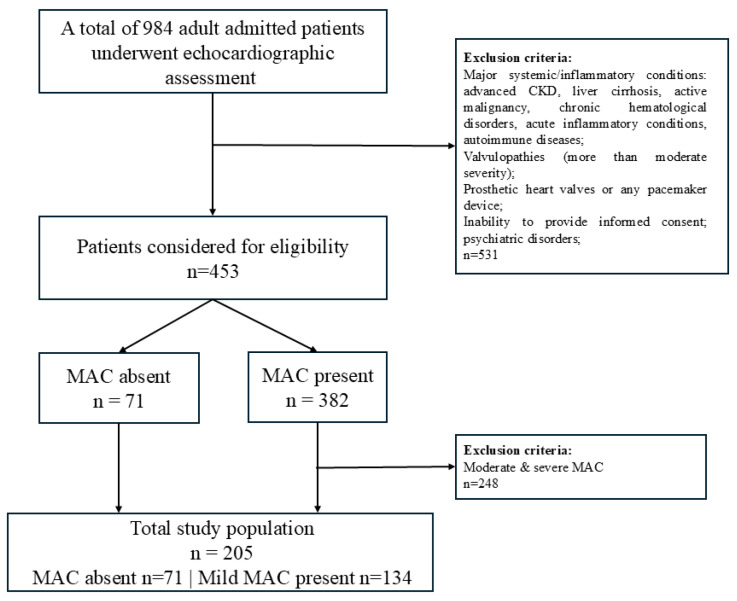
Flowchart of patient enrollment and application of exclusion criteria.

After applying the inclusion and exclusion criteria, 205 patients were retained for analysis, comprising 134 individuals with mild MAC and 71 individuals without MAC, who formed the MAC and non-MAC groups (Figure 1). The results below summarize clinical and cardiometabolic characteristics, followed by the relationship between MAC and inflammatory indices.

### 3.1. Baseline Characteristics of the Study Population

Baseline clinical, cardiovascular, cardiometabolic, echocardiographic, and laboratory characteristics stratified by MAC status are summarized in Table 1. Patients with MAC were significantly older than those without MAC (66.5 vs. 58.0 years, *p* < 0.001) and exhibited a higher systolic BP (149.1 ± 22.3 mmHg vs. 138.1 ± 17.8 mmHg, *p* < 0.001), as well as a marked prevalence of arterial hypertension (96.3% vs. 69.0%, *p* < 0.001). Measures of adiposity, including waist circumference (103.0 cm vs. 97.0 cm, *p* = 0.021) and BMI (30.7 ± 5.5 kg/m^2^ vs. 28.7 ± 5.3 kg/m^2^, *p* = 0.013) were also significantly greater in the MAC group, while sex distribution and smoking status did not differ between groups (*p* = 0.203 and *p* = 0.365, respectively).

From a cardiometabolic perspective, disturbances in glucose metabolism were more frequent among patients with MAC, with a higher prevalence of both prediabetes (54.5% vs. 43.7%, *p* = 0.004) and T2D (16.4% vs. 5.6%, *p* = 0.004). Heart failure was substantially more common in the MAC group (91% vs. 53.5%, *p* < 0.001), whereas the prevalence of coronary artery disease and atrial fibrillation was comparable between groups.

Echocardiographic assessment revealed significant differences in patients with MAC, including increased interventricular septum thickness (*p* = 0.049), larger left atrial volume (*p* < 0.001), greater right atrial diameter (*p* = 0.030), and increased right ventricular basal diameter (*p* = 0.014). LVEF did not differ between groups, while left ventricular volumes showed only borderline differences.

Laboratory analyses demonstrated higher levels of HbA1c (5.85% vs. 5.60%, *p* < 0.001), triglycerides (113.5 mg/dL vs. 81.0 mg/dL, *p* < 0.001), and NT-proBNP (116.0 pg/mL vs. 85.0 pg/mL, *p* = 0.031) in the MAC group, accompanied by lower HDL-cholesterol concentrations (45.0 mg/dL vs. 53.0 mg/dL, *p* < 0.001). Renal function, assessed by eGFR, as well as LDL-cholesterol and global hematological parameters, including hemoglobin, total leukocyte count, and platelet count, were similar between groups.

**Figure 2:**
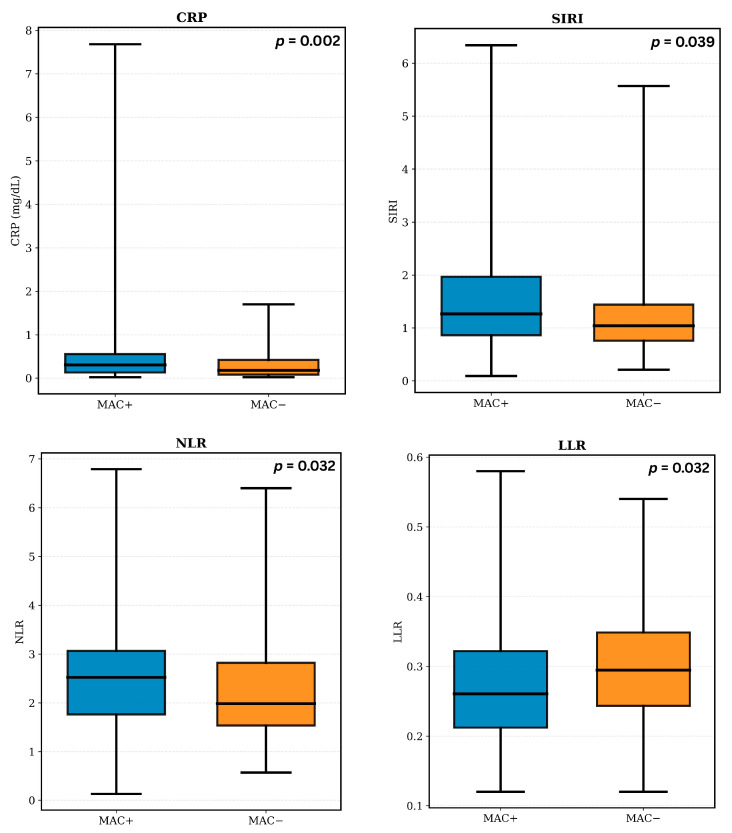
Distribution of hematological inflammatory markers by MAC status.

Inflammatory indices (Figure 2) showed modest but statistically significant differences between patients with and without MAC. Patients with MAC exhibited higher NLR (2.52 vs. 1.99, *p* = 0.032) and SIRI values (1.27 vs. 1.04, *p* = 0.039) and lower LLR values compared to those without MAC (0.26 vs. 0.29, *p* = 0.032). In parallel, serum CRP levels were also elevated in the MAC group (0.31 mg/dL vs. 0.18 mg/dL, *p* = 0.002), whereas NMR, PLR, and SII did not differ significantly between groups.

### 3.2. ROC Analysis of Hematological Inflammatory Markers

**Figure 3:**
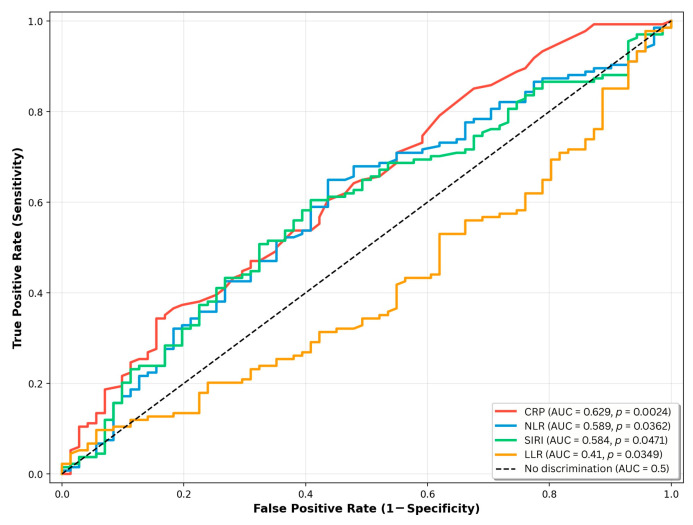
ROC curves for inflammatory markers. An AUC below 0.5 for LLR indicates inverse discrimination, with lower values observed in patients with MAC.

In ROC curve analysis (Figure 3), CRP demonstrated the highest AUC at 0.629 (95% CI: 0.543–0.713), followed by NLR (AUC 0.589; 95% CI: 0.503–0.672) and SIRI (AUC 0.584; 95% CI: 0.500–0.668). In contrast, the LLR displayed an AUC below 0.5 (0.419; 95% CI: 0.330–0.493), consistent with lower values observed in the MAC group.

### 3.3. Inflammatory Markers in Relation to Clinical and Cardiometabolic Variables

Because inflammatory indices may be influenced by age, adiposity, glucose metabolism, cardiac dysfunction, and renal impairment, we further explored their relationship with selected clinical and biochemical variables.

Correlations between inflammatory markers and selected clinical variables are presented in Table 2. Spearman correlation analysis showed statistically significant associations between age and all four inflammatory markers, including CRP, NLR, LLR, and SIRI. CRP demonstrated significant correlations with BMI, waist circumference, and NT-proBNP levels. NLR was weakly correlated with eGFR, while LLR showed a significant positive correlation with eGFR and a negative correlation with age. SIRI was significantly correlated with age, NT-proBNP, and eGFR.

Given the known influence of glucose metabolism on inflammatory markers, we further examined their distribution according to glycemic status (Table 3). Median values and interquartile ranges of CRP, NLR, LLR, and SIRI are shown for participants without dysglycemia, with prediabetes, and with T2D. Statistically significant differences across glycemic categories were observed for CRP, NLR, and SIRI, whereas LLR showed no significant overall differences between groups.

To assess whether the associations observed in univariable analyses persisted after accounting for relevant clinical and cardiometabolic factors, multivariable logistic regression models were constructed with MAC status as the dependent variable (Table 4, Table 5, Table 6 and Table 7). Each model included a single inflammatory marker (CRP, NLR, LLR, and SIRI) together with clinically relevant covariates selected a priori, including age, measures of adiposity, glucose metabolism status, NT-proBNP levels, and renal function, as applicable.

After multivariable adjustment, none of the inflammatory markers demonstrated a statistically significant association with the presence of MAC. In contrast, age remained significantly associated with MAC in the NLR-, LLR-, and SIRI-based models, with ORs ranging from 1.056 to 1.063 per year increase (all *p* ≤ 0.001). Abnormalities of glucose metabolism were consistently associated with MAC, with prediabetes showing ORs between 2.43 and 3.63 and T2D showing ORs between 5.91 and 6.19 across applicable models (all *p* ≤ 0.004). Renal function, expressed as eGFR, was additionally associated with MAC in the NLR- and SIRI-based models (OR 1.03 per 1 mL/min/1.73 m^2^ increase; *p* = 0.022 and *p* = 0.029, respectively).

## 4. Discussion

In this cross-sectional study of patients with cardiometabolic risk factors, several inflammatory biomarkers derived from routine hematological testing (including CRP, NLR, SIRI, and LLR) showed significant differences between patients with and without mild MAC in unadjusted analyses. However, these associations were no longer evident after multivariable adjustment for age, abnormalities of glucose metabolism, and other relevant cardiometabolic covariates. By contrast, age and disturbances in glucose metabolism, particularly prediabetes and T2D, remained robustly and independently associated with the presence of mild MAC.

The observed pattern of results is compatible with at least three interpretations, which our cross-sectional design cannot distinguish. First, inflammatory markers may primarily reflect the cardiometabolic burden (particularly dysglycemia, obesity, and their metabolic consequences) that accompanies MAC, rather than representing MAC-specific inflammatory processes. This interpretation is supported by the consistent associations we observed between inflammatory indices and cardiometabolic variables and by the strong independent associations of MAC with age and glucose metabolism disorders. Second, both MAC and increased levels of inflammatory markers may arise as parallel consequences of shared cardiometabolic derangements, particularly chronic hyperglycemia and insulin resistance, rather than reflecting a direct causal relationship between inflammation and MAC. Third, the absence of independent associations in multivariable models may be explained by limited statistical power to detect small, independent inflammatory effects on MAC after adjustment for strongly correlated cardiometabolic variables. The relatively modest between-group differences in inflammatory markers suggest that any independent contribution of inflammation, if present, is likely minor and would require larger study populations to be reliably identified.

Chronic low-grade inflammation is a well-recognized feature of T2D and the broader cardiometabolic syndrome, conditions that are strongly age-dependent and characterized by sustained exposure to hyperglycemia, excess free fatty acids, and insulin resistance [22,23]. These metabolic disturbances promote inflammatory signaling within the vascular wall and have been consistently linked to both arterial and valvular calcification [23,24]. Experimental and human studies suggest that in this metabolic context, vascular smooth muscle cells undergo phenotypic changes resembling endochondral ossification, with loss of calcification inhibitors and acquisition of osteochondrogenic markers [23,25].

Importantly, accumulating evidence indicates that hyperglycemia may also promote calcific remodeling through local, tissue-level mechanisms, such as advanced glycation end-product accumulation, oxidative stress, and osteogenic signaling [26]. In addition to hyperglycemia, insulin resistance may further contribute to calcific remodeling by attenuating protective metabolic signaling pathways. Under physiological conditions, insulin exerts anti-calcific effects through the regulation of calcification inhibitors; these mechanisms are impaired in insulin-resistant states, favoring osteogenic signaling and phenotypic transformation of the interstitial cells [27]. Such metabolic alterations provide a plausible link between abnormalities of glucose metabolism and valvular calcification that may occur independently of circulating systemic inflammatory markers.

Our results situate MAC within the broader continuum of metabolically driven calcific conditions. The consistent and robust associations between MAC and abnormalities of glucose metabolism—particularly prediabetes and T2D—across all analytical models, together with the attenuation of inflammatory marker associations after multivariable adjustments, support the interpretation that MAC represents a manifestation of chronic metabolic dysfunction, with inflammation reflecting an accompanying feature of the underlying metabolic milieu rather than an independent driver [28].

Our results both confirm and build upon previous observations in the literature. Varol et al. reported an association between NLR and MAC in a small cohort, demonstrating that NLR was significantly elevated in patients with MAC [10]. Yayla et al. reported that PLR was significantly higher in MAC and independently associated with MAC after adjusting for conventional risk factors such as age, hypertension, LDL-cholesterol, and triglycerides [19]. In our study population, inflammatory biomarkers differed between groups in crude analyses; however, these associations were no longer evident after thorough adjustment for cardiometabolic confounders. The divergence from previous reports may be attributed to the strict population selection, restriction to mild MAC, and extensive cardiometabolic adjustment, which together may have attenuated the independent inflammatory signal. Furthermore, the relatively limited sample size may have reduced the ability to detect modest inflammatory effects once full adjustment was performed. Taken together, these factors indicate that the relationship between inflammatory markers and MAC may be complex and may vary according to disease severity, population characteristics, and the extent of underlying cardiometabolic dysfunction.

A strength of this study is its prospective design, with systemic echocardiographic and laboratory assessment of consecutively evaluated patients, allowing consistent characterization of their cardiometabolic profile. Nevertheless, several limitations should be acknowledged. First of all, the cross-sectional, single-center design in a tertiary-care referred population precludes any causal inference regarding the temporal relationship between inflammation and MAC and may introduce potential selection bias, limiting generalizability to broader settings. Whether elevated inflammatory indices precede MAC development or simply reflect the underlying cardiometabolic milieu cannot be determined in this cross-sectional study and requires longitudinal investigation. Second, the strict exclusion criteria and deliberate focus on mild MAC mean that patients with advanced MAC or major inflammatory comorbidities were not represented, so the findings may not apply across the full spectrum of MAC. Third, the unequal distribution of patients with and without MAC, inherent to the consecutive enrollment strategy, may have limited the statistical power to detect modest independent associations after multivariable adjustment. Although propensity score matching could have been considered, its application would have substantially reduced the effective sample size and restricted the analysis to a highly selected subgroup. Given the cross-sectional, observational design, the absence of an assigned exposure or intervention and the study’s focus on association rather than causality, multivariable regression was considered the most appropriate analytical approach.

The loss of significance of inflammatory markers after adjustment highlights the predominant influence of age and impaired glucose metabolism in mild MAC. Although low-grade inflammation may plausibly mediate the link between dysglycemia and MAC, formal mediation analysis was not undertaken due to the cross-sectional study design, which precludes reliable temporal inference. Moreover, the inflammatory indices assessed are indirect hematological surrogates rather than specific molecular mediators, limiting their appropriateness for mediation modeling. Accordingly, the focus of this study was on independent associations rather than causal or mechanistic pathways. Our approach was designed to capture predominantly low-grade, subclinical inflammation using simple hematological indices derived from the routine complete blood count; however, we did not assess cytokines or other molecular inflammatory markers. Therefore, the absence of independent associations should be interpreted in the context of early-stage MAC within a cardiometabolic population.

## 5. Conclusions

In this cross-sectional study of patients with cardiometabolic risk factors and mild MAC, we found that while inflammatory indices showed unadjusted differences between patients with and without MAC, these associations did not persist after accounting for age and metabolic factors, particularly glucose metabolism abnormalities. These findings are most compatible with inflammatory markers primarily reflecting the cardiometabolic milieu in which MAC occurs. Age and disturbances in glucose metabolism emerged as the dominant factors independently associated with MAC, reinforcing the importance of metabolic dysfunction in MAC pathogenesis. Whether targeted anti-inflammatory strategies beyond optimal metabolic control might prevent or modify MAC development remains an important unanswered question requiring prospective interventional studies.

## Figures and Tables

**Table 1:** General clinical, biological, and echocardiographic characteristics of patients with (MAC+) and without MAC (MAC−).

Parameter	MAC+ (n = 134)	MAC− (n = 71)	Total (n = 205)	Statistical Test	*p*-Value
Clinical and anthropometric characteristics					
Age (years)	66.5 (56.0–73.0)	58.0 (50.0–69.0)	65.0 (54.0–72.0)	Mann-Whitney U = 3351.0	<0.001 *
Sex (female)	90 (67.2%)	38 (53.5%)	128 (59.5%)	χ^2^ = 1.618	0.203
Systolic BP (mmHg)	149.1 ± 22.3	138.1 ± 17.8	145.3 ± 21.4	t = 3.858	<0.001 *
Arterial Hypertension	129 (96.3%)	49 (69.0%)	178 (86.8%)	χ^2^ = 30.145	<0.001 *
Waist circumference (cm)	103.0 (92.0–114.8)	97.0 (89.0–108.0)	102.0 (92.0–112.0)	Mann-Whitney U = 3822.5	0.021 *
BMI (kg/m^2^)	30.7 ± 5.5	28.7 ± 5.3	30.0 ± 5.5	t = 2.520	0.013 *
Smoking status	52 (38.8%)	23 (32.4%)	75 (36.6%)	χ^2^ = 0.822	0.365
Cardiovascular and metabolic comorbidities					
Hepatic steatosis	98 (66.4%)	41 (57.7%)	130 (63.4%)	χ^2^ = 1.504	0.220
Prediabetes	73 (54.5%)	31 (43.7%)	104 (50.7%)	χ^2^ = 11.244	0.004 *
Type 2 Diabetes	22 (16.4%)	4 (5.6%)	26 (12.7%)	χ^2^ = 11.244	0.004 *
Heart failure	122 (91%)	38 (53.5%)	172 (83.9%)	χ^2^ = 74.231	<0.001 *
NYHA II–III	115 (85.8%)	38 (53.5%)	153 (74.6%)	χ^2^ = 79.414	<0.001 *
Coronary artery disease	35 (26.1%)	16 (22.5%)	51 (24.9%)	χ^2^ = 0.319	0.572
Atrial fibrillation	32 (23.9%)	10 (14.1%)	42 (20.5%)	χ^2^ = 3.556	0.314
Echocardiographic characteristics					
IVS (mm)	11.0 (10.0–12.0)	11.0 (9.0–12.0)	11.0 (10.0–12.0)	Mann-Whitney U = 3977.5	0.049 *
PWT (mm)	11.0 (10.0–12.0)	11.0 (9.0–12.0)	11.0 (10.0–12.0)	Mann-Whitney U = 4190.5	0.150
LVEDD (mm)	41.0 (37.0–45.0)	41.0 (36.0–46.0)	41.0 (37.0–45.0)	Mann-Whitney U = 4553.0	0.613
LVEDV (mL/m^2^)	81.5 (69.8–97.0)	76.0 (63.0–95.0)	80.0 (67.5–96.5)	Mann-Whitney U = 4022.0	0.069 +
LVESV (mL/m^2^)	35.0 (23.0–43.5)	28.0 (23.0–38.0)	32.0 (23.0–42.0)	Mann-Whitney U = 4009.0	0.064 +
LVEF (%)	59.0 (54.0–65.0)	60.0 (55.0–64.0)	60.0 (55.0–65.0)	Mann-Whitney U = 4278.0	0.233
LA volume (mL/m^2^)	82.0 (65.8–101.3)	70.0 (58.0–84.0)	76.0 (62.0–97.5)	Mann-Whitney U = 3390.5	<0.001 *
RA diameter (mm)	58.0 (44.0–70.0)	50.0 (41.0–61.0)	55.0 (42.0–67.0)	Mann-Whitney U = 3880.0	0.030 *
RV basal diameter (mm)	35.0 (32.0–38.3)	33.0 (29.0–37.0)	34.0 (32.0–38.0)	Mann-Whitney U = 3769.5	0.014 *
Laboratory characteristics					
CRP (mg/dL)	0.31 (0.13–0.56)	0.18 (0.08–0.42)	0.28 (0.12–0.5)	Mann-Whitney U = 3518.500	0.002 *
Glucose (mg/dL)	100.5 (90.75–113)	97.0 (86.0–105.0)	99.0 (88.0–110.0)	Mann-Whitney U = 4031.5	0.073 +
HbA1c (%)	5.85 (5.60–6.10)	5.60 (5.30–5.90)	5.80 (5.40–6.00)	Mann-Whitney U = 3306.5	<0.001 *
LDL–cholesterol (mg/dL)	123.51 ± 47.93	128.99 ± 43.95	125.41 ± 46.56	t = −0.800	0.425
HDL–cholesterol (mg/dL)	45.0 (37.0–54.0)	53.0 (42.0–61.0)	47.0 (39.0–56.0)	Mann-Whitney U = 3390.5	<0.001 *
Triglycerides (mg/dL)	113.5 (85.0–159.3)	81.0 (63.0–133.0)	106.0 (75.0–146.0)	Mann-Whitney U = 3468.0	<0.001 *
NT-proBNP (pg/mL)	116.0 (61.51–353.25)	85.0 (34.0–185.0)	102.0 (50.4–290.5)	Mann-Whitney U = 3885.5	0.031 *
eGFR (mL/min/1.73 m^2^)	86.7 (72.0–97.2)	87.5 (73.3–101.4)	87.4 (72.9–98.4)	Mann-Whitney U = 4354.5	0.319
Hemoglobin (g/dL)	14 (13.2–14.9)	13.6 (13.2–14.3)	13.9 (13.2–14.7)	Mann-Whitney U = 4241.500	0.202
Leukocytes (×10^3^/μL)	6.8 (5.8–8.2)	6.8 (5.3–7.9)	6.8 (5.7–8.1)	Mann-Whitney U = 4372.000	0.341
Neutrophils (×10^3^/μL)	4.2 (3.4–5.6)	4.1 (2.9–5)	4.1 (3.3–5.4)	Mann-Whitney U = 4134.000	0.123
Neutrophils (%)	63.3 (56–67.5)	60.2 (52.7–66.3)	61.8 (55.1–66.7)	Mann-Whitney U = 4067.500	0.088 +
Monocytes (×10^3^/μL)	0.55 (0.43–0.69)	0.51 (0.42–0.63)	0.52 (0.43–0.67)	Mann-Whitney U = 4375.500	0.345
Lymphocytes (×10^3^/μL)	1.74 (1.45–2.17)	1.84 (1.49–2.34)	1.75 (1.46–2.21)	Mann-Whitney U = 4308.500	0.267
Lymphocytes (%)	25.9 (21.8–31.9)	29 (23.2–34.9)	26.5 (22.3–33.4)	Mann-Whitney U = 4014.000	0.066 +
Platelets (×10^3^/μL)	236.8 ± 60.2	240.9 ± 61.0	238.2 ± 60.4	t = −0.462	0.645
Hematological inflammatory parameters					
NLR	2.52 (1.77–3.07)	1.99 (1.54–2.82)	2.4 (1.67–2.95)	Mann-Whitney U = 3963.000	0.032 *
NMR	7.68 (6.32–9.55)	7.35 (6.16–9.47)	7.52 (6.32–9.54)	Mann-Whitney U = 4488.000	0.506
LLR	0.26 (0.21–0.32)	0.29 (0.24–0.35)	0.27 (0.23–0.33)	Mann-Whitney U = 3889.000	0.032 *
PLR	127.78 (103.79–166.15)	130.32 (99.73–146.96)	128.46 (102.85–159.96)	Mann-Whitney U = 4557.500	0.622
SIRI	1.27 (0.86–1.97)	1.04 (0.76–1.44)	1.19 (0.85–1.75)	Mann-Whitney U = 3922.000	0.039 *
SII	68.39 (52.44–92.11)	66.21 (52.13–79.02)	67.62 (52.41–85.83)	Mann-Whitney U = 4263.000	0.222

BP: blood pressure; BMI: body mass index; eGFR: estimated glomerular filtration rate; NYHA: New York Heart Association; IVS: interventricular septum; PWT: posterior wall thickness; LVEDD: left ventricular end-diastolic diameter; LVEDV: left ventricular end-diastolic volume; LVESV: left ventricular end-systolic volume; LVEF: left ventricular ejection fraction; LA: left atrium; RA: right atrium; RV: right ventricle; CRP: C-reactive protein; HbA1c: glycated hemoglobin; HDL: high-density lipoprotein; LDL: low-density lipoprotein; NT-proBNP: N-terminal pro-B-type natriuretic peptide; NLR: Neutrophil-to-Lymphocyte Ratio; NMR: Neutrophil-to-Monocyte Ratio; LLR: Lymphocyte-to-Leukocyte Ratio; PLR: Platelet-to-Lymphocyte Ratio; SIRI: Systemic Inflammation Response Index; SII: Systemic Immune-Inflammation Index. + Trend toward significance. * Statistical significance.

**Table 2:** Spearman correlations between inflammatory markers and clinical variables.

Variable	CRP, ρ (*p*)	NLR, ρ (*p*)	LLR, ρ (*p*)	SIRI, ρ (*p*)
Age	0.162 (0.020 *)	0.177 (0.011 *)	−0.191 (0.006 *)	0.184 (0.008 *)
BMI	0.322 (<0.001 *)	−0.008 (0.908)	0.026 (0.706)	−0.054 (0.438)
Waist circumference	0.297 (<0.001 *)	0.013 (0.849)	−0.003 (0.967)	−0.008 (0.914)
NT-proBNP	0.260 (<0.001 *)	0.081 (0.247)	−0.110 (0.116)	0.151 (0.031 *)
eGFR	−0.102 (0.145)	−0.137 (0.050 *)	0.147 (0.036 *)	−0.138 (0.048 *)

CRP: C-reactive protein; NLR: neutrophil-to-lymphocyte ratio; LLR: lymphocyte-to-leukocyte ratio; SIRI: Systemic Inflammation Response Index; BMI: Body Mass Index; NT-proBNP: N-terminal pro-B-type natriuretic peptide; eGFR: estimated glomerular filtration rate. * Statistically significant *p*-values (*p* ≤ 0.05).

**Table 3:** Inflammatory markers according to glucose metabolism status.

Marker	No Dysglycemia M ± SD Median (IQR)	Prediabetes M ± SD Median (IQR)	Type 2 Diabetes M ± SD Median (IQR)	H (Kruskal-Wallis)	*p*-Value
CRP	0.374 ± 0.790 0.170 (0.090 ÷ 0.430)	0.624 ± 1.248 0.320 (0.130 ÷ 0.517)	0.475 ± 0.460 0.395 (0.187 ÷0.570)	7.008	0.030 *
NLR	2.315 ± 1.141 2.055 (1.553 ÷ 2.691)	2.580 ± 1.248 2.411 (1.737 ÷ 2.937)	2.863 ± 1.132 2.998 (2.338 ÷ 3.456)	7.329	0.026 *
LLR	0.293 ± 0.085 0.288 (0.240 ÷ 0.348)	0.276 ± 0.084 0.266 (0.224 ÷ 0.327)	0.251 ± 0.084 0.235 (0.200 ÷ 0.272)	5.591	0.061
SIRI	1.223 ± 0.810 0.962 (0.759 ÷ 1.39)	1.567 ± 1.117 1.211 (0.866 ÷ 1.982)	1.804 ± 0.902 1.629 (1.199 ÷ 2.584)	7.320	0.026 *

CRP: C-reactive protein; NLR: neutrophil-to-lymphocyte ratio; LLR: lymphocyte-to-leukocyte ratio; SIRI: Systemic Inflammation Response Index. * Statistically significant *p*-values (*p* < 0.05).

**Table 4:** Multivariable logistic regression models for the association between CRP and MAC.

Variable	B (β)	OR (95% CI)	*p* Value
CRP	1.162	3.197 (0.835–12.240)	*p* = 0.090
Age (per year)	−0.005	0.995 (0.956–1.036)	*p* = 0.807
BMI (per kg/m^2^)	−0.014	0.986 (0.851–1.142)	*p* = 0.851
Waist circumference	−0.022	0.979 (0.936–1.023)	*p* = 0.337
Prediabetes vs. none	0.888	2.430 (1.036–5.698)	*p* = 0.041 *
Type 2 diabetes vs. none	1.776	5.906 (1.520–22.937)	*p* = 0.010 *
NT-proBNP (per 1 pg/mL)	−0.001	0.999 (0.999–1.000)	*p* = 0.099

CRP: C-reactive protein; BMI: body mass index; NT-proBNP: N-terminal pro-B-type natriuretic peptide; MAC: Mitral annular calcification. * Statistically significant *p*-values.

**Table 5:** Multivariable logistic regression models for the association between NLR and MAC.

Variable	B (β)	OR (95% CI)	*p* Value
NLR	0.171	1.186 (0.900–1.564)	*p* = 0.225
Age (per year)	0.055	1.056 (1.023–1.090)	*p* = 0.001 *
Prediabetes vs. none	1.284	3.610 (1.787–7.291)	*p* < 0.001 *
Type 2 diabetes vs. none	1.818	6.160 (2.012–18.866)	*p* = 0.001 *
eGFR (per unit increase)	0.028	1.029 (1.004–1.054)	*p* = 0.022 *

NLR: neutrophil-to-lymphocyte ratio; eGFR: estimated glomerular filtration rate; MAC: Mitral annular calcification. * Statistically significant *p*-values.

**Table 6:** Multivariable logistic regression models for the association between LLR and MAC.

Variable	B (β)	OR (95% CI)	*p* Value
LLR	−2.931	0.053 (0.001–2.008)	*p* = 0.113
Age (per year)	0.061	1.063 (1.031–1.096)	*p* < 0.001 *
eGFR (per unit increase)	0.020	1.020 (0.997–1.043)	*p* = 0.083

LLR: lymphocyte-to-leukocyte ratio; eGFR: estimated glomerular filtration rate; MAC: Mitral annular calcification. * Statistically significant *p*-values.

**Table 7:** Multivariable logistic regression models for the association between SIRI and MAC.

Variable	B (β)	OR (95% CI)	*p* Value
SIRI	0.127	1.136 (0.812–1.589)	*p* = 0.458
Age (per year)	0.056	1.057 (1.023–1.092)	*p* = 0.001 *
Prediabetes vs. none	1.288	3.625 (1.791–7.336)	*p* < 0.001 *
Type 2 diabetes vs. none	1.823	6.189 (2.023–18.935)	*p* < 0.001 *
NT-proBNP (per 1 pg/mL)	0.000	1.000 (0.999–1.001)	*p* = 0.637
eGFR (per unit increase)	0.027	1.027 (1.003–1.052)	*p* = 0.029 *

SIRI: Systemic Inflammation Response Index; eGFR: estimated glomerular filtration rate; NT-proBNP: N-terminal pro-B-type natriuretic peptide; MAC: Mitral annular calcification. * Statistically significant *p*-values.

## Data Availability

Data is contained within the article.

## Abbreviations

The following abbreviations are used in this manuscript:

AUC	Area Under the Curve
BMI	Body Mass Index
BP	Blood Pressure
CI	Confidence Interval
CRP	C-Reactive Protein
EDTA	Ethylenediaminetetraacetic Acid
HbA1c	Hemoglobin A1c (Glycated Hemoglobin)
hs-CRP	High-Sensitivity C-Reactive Protein
IQR	Interquartile Range
LLR	Lymphocyte-to-Leukocyte Ratio
LVEF	Left Ventricular Ejection Fraction
MAC	Mitral Annular Calcification
NLR	Neutrophil-to-Lymphocyte Ratio
NMR	Neutrophil-to-Monocyte Ratio
NT-proBNP	N-Terminal Pro-B-Type Natriuretic Peptide
NYHA	New York Heart Association
PLR	Platelet-to-Lymphocyte Ratio
ROC	Receiver Operating Characteristic
SD	Standard Deviation
SIRI	Systemic Inflammation Response Index
SII	Systemic Immune-Inflammation Index
SI	Systemic Inflammation
SPSS	Statistical Package for the Social Sciences
T2D	Type 2 Diabetes
